# Biomarkers of Inflammation and Their Association With the Severity and Onset of Preeclampsia: A Systematic Review

**DOI:** 10.7759/cureus.87734

**Published:** 2025-07-11

**Authors:** Rumaissa Haidar Abdeldaem Mohamed, Nahla Mohamed Ahmed Ali Alfaki, Rania E Belal, Azza Mohamed Ali Dawelbait, Reem Badawi Hamad Yousif, Shahinaz A. E Mohamed, Hind Suliman Badre Adam, Eman Mohammed Abbashar Abdelmahmoud

**Affiliations:** 1 Obstetrics and Gynaecology, Abu Arish General Hospital, Jazan, SAU; 2 Obstetrics and Gynaecology, Mouwasat Hospital, Jubail, SAU; 3 Obstetrics and Gynaecology, Portiuncula University Hospital, Galway, IRL; 4 Gynaecologic Oncology, Sheikh Khalifa Specialty Hospital, Ras Al Khaimah, ARE; 5 Obstetrics and Gynaecology, Royal Surrey County Hospital, Surrey, GBR; 6 Obstetrics and Gynaecology, Bidiyah Hospital, Bidiya, OMN; 7 Obstetrics and Gynaecology, Sultan Qaboos Hospital, Salalah, OMN

**Keywords:** cytokines, disease severity, inflammatory biomarkers, maternal health, preeclampsia, systematic review

## Abstract

Preeclampsia (PE) remains a leading cause of maternal and perinatal morbidity and mortality, with systemic inflammation playing a central role in its pathogenesis. Despite extensive research on inflammatory biomarkers, inconsistencies persist regarding their associations with disease severity and onset. This systematic review synthesizes current evidence on the relationship between inflammatory biomarkers and PE, focusing on their diagnostic and prognostic potential. Following the Preferred Reporting Items for Systematic Reviews and Meta-Analyses 2020 guidelines, a comprehensive search was conducted across five databases (PubMed, Scopus, Web of Science, Embase, and CINAHL) to identify observational studies investigating inflammatory biomarkers in PE. Eligible studies included case-control, cross-sectional, and cohort designs with normotensive controls. Data extraction covered study characteristics, biomarker profiles, and clinical outcomes. Methodological quality was assessed using the Newcastle-Ottawa Scale. In total, 13 studies were included, predominantly from diverse geographical regions. Pro-inflammatory cytokines and acute-phase proteins (C-reactive protein) were consistently elevated in PE, with distinct profiles for early-onset (placental-driven inflammation) and late-onset (systemic inflammation) subtypes. Biomarkers such as neopterin and soluble urokinase-type plasminogen activator receptor showed promise in stratifying disease severity. Maternal-fetal inflammatory cascades were evident, with correlations between maternal biomarkers and adverse neonatal outcomes. However, heterogeneity in study designs, biomarker measurement timing, and inconsistent adjustments for confounders limited comparability. Quality assessment revealed seven low-risk and six moderate-risk studies, with no high-risk bias. Inflammatory biomarkers demonstrate significant associations with PE severity and onset, supporting their role in disease monitoring and risk stratification. However, methodological inconsistencies highlight the need for standardized protocols and larger, longitudinal studies to validate their clinical utility. Future research should integrate multi-omics approaches to refine biomarker panels and elucidate causal pathways, ultimately guiding targeted interventions.

## Introduction and background

Preeclampsia (PE) remains one of the most significant obstetric complications worldwide, affecting approximately 5-10% of pregnancies and contributing substantially to maternal and perinatal morbidity and mortality [1]. This multisystem disorder, characterized by new-onset hypertension and proteinuria after 20 weeks of gestation, represents not merely a pregnancy-specific condition but rather a complex syndrome with diverse pathophysiological pathways [2]. Among these pathways, the role of systemic inflammation has emerged as a central focus in contemporary research, with growing evidence suggesting that inflammatory processes may serve as both mediators and markers of disease progression [3]. The physiological adaptations of normal pregnancy already involve a state of controlled inflammation, but in PE, this balance appears disrupted, leading to excessive systemic inflammatory responses that contribute to endothelial dysfunction and the characteristic clinical manifestations of the disorder [4].

The investigation of inflammatory biomarkers in PE has gained considerable momentum in recent years, driven by the need for better predictive tools and a deeper understanding of disease mechanisms [5]. Cytokines, acute-phase proteins, and other mediators of inflammation have been extensively studied in this context, with particular attention to their potential roles in differentiating disease subtypes and predicting clinical outcomes. Interleukins (ILs) such as IL-6 and tumor necrosis factor-alpha (TNF-α), along with C-reactive protein (CRP) and various adhesion molecules, have shown promise in reflecting the inflammatory milieu associated with PE [6]. However, the existing literature presents considerable heterogeneity in findings, with variations in biomarker profiles observed across different study populations, gestational ages, and disease severities [7]. This inconsistency may stem from methodological differences, including variations in sample timing, assay techniques, and case definitions, as well as the inherent biological complexity of the condition itself.

The relationship between inflammatory biomarkers and PE severity represents a particularly important area of investigation [5,8]. Emerging evidence suggests that distinct inflammatory signatures may characterize different clinical presentations of the disease, from early-onset severe forms to late-onset mild cases [9]. The potential to stratify patients based on biomarker profiles could have significant clinical implications, enabling more targeted monitoring and intervention strategies [10]. Furthermore, understanding these inflammatory patterns may provide insights into the underlying pathophysiological differences between PE subtypes, which currently remain incompletely understood. The temporal aspects of biomarker expression also warrant careful consideration, as the inflammatory cascade in PE likely evolves throughout the course of the disease, with different mediators becoming prominent at various stages of disease progression [11].

Despite numerous studies examining inflammatory markers in PE [5-7,11], there remains a need for a comprehensive synthesis of this evidence. Previous systematic reviews have often focused on specific biomarkers or limited aspects of the inflammatory response, leaving gaps in our understanding of how these markers collectively relate to disease characteristics. Moreover, the quality and methodological rigor of existing studies vary considerably, necessitating careful evaluation of potential biases and limitations in the evidence base. This systematic review addresses these needs by comprehensively examining the association between inflammatory biomarkers and PE severity and onset, while critically appraising the methodological quality of included studies.

## Review

Methodology

Design and Objective

This systematic review was conducted following the Preferred Reporting Items for Systematic Reviews and Meta-Analyses (PRISMA) 2020 guidelines to ensure methodological rigor and transparency [12]. The objective was to synthesize existing evidence on the association between inflammatory biomarkers and the severity or onset of PE.

Eligibility Criteria

Studies were included if they met the following criteria: they were observational in design (case-control, cross-sectional, or cohort studies) and investigated inflammatory biomarkers in relation to PE; they reported on biomarker levels in connection with PE severity (such as mild versus severe or early-onset versus late-onset) or onset; they included a control group (either normotensive pregnant women or non-pregnant controls); and they were published as full-text articles in English. Excluded from the review were studies such as review articles, editorials, conference abstracts, animal studies, and those that did not measure biomarkers relevant to inflammation.

Search Strategy

A comprehensive and systematic search was conducted across the following five major electronic databases: PubMed, Scopus, Web of Science, Embase, and CINAHL, covering literature from their inception up to June 5, 2025. The search strategy incorporated a combination of Medical Subject Headings (MeSH) terms and free-text keywords to capture all relevant studies. Key terms included variations of “preeclampsia,” “pre-eclampsia,” “gestational hypertension,” and “toxemia” for the condition, alongside terms like “inflammation,” “inflammatory markers,” “cytokines,” “interleukins,” “TNF-alpha,” and “CRP” for the biomarkers. The search strategy for each database is included in the Appendices. No restrictions were placed on publication dates or study designs to ensure a broad and inclusive search; however, the studies included in this review were published between 2010 and 2025.

Study Selection Process

The study selection process adhered to the PRISMA 2020 flow diagram to ensure transparency and reproducibility. After removing duplicate records, two independent reviewers (HSBA and EMAA, both authors of this review) screened the titles and abstracts of the remaining studies to assess their relevance. Full-text articles of potentially eligible studies were then retrieved and evaluated against the predefined inclusion criteria. Any disagreements between the reviewers during the screening process were resolved through discussion or, if necessary, by consulting a third author (RBHY) who acted as a tiebreaker. Detailed reasons for excluding studies at the full-text stage were documented to maintain clarity and accountability in the selection process.

Data Extraction

A standardized data extraction form was developed to systematically collect key information from each included study. Two reviewers independently extracted data on study characteristics such as the first author, publication year, country, study design, and sample size. Population details, including the criteria used to define PE, gestational age, and severity classification, were also recorded. Biomarker-related data encompassed the types of biomarkers measured, the timing of their measurement, and the assay methods employed. Key findings, such as associations between biomarkers and PE severity or onset, along with relevant effect estimates and their statistical significance, were extracted. Discrepancies in data extraction were resolved through consensus to ensure accuracy.

Quality Assessment

The methodological quality of the included studies was evaluated using the Newcastle-Ottawa Scale (NOS), a validated tool for assessing the risk of bias in observational studies [13]. The NOS evaluates three critical domains, namely, selection, comparability, and outcome. The selection domain assesses the representativeness of cases, the selection of controls, and the ascertainment of exposure. The comparability domain examines whether the studies controlled for confounding factors such as age, body mass index (BMI), and gestational age. The outcome domain evaluates the assessment of outcomes and the adequacy of follow-up. Each study was assigned a score ranging from 0 to 9, with higher scores indicating a lower risk of bias. Studies were categorized as low (7-9 stars), moderate (5-6 stars), or high risk (<5 stars) based on their scores. Two reviewers independently conducted the quality assessments, and any disagreements were resolved through discussion to ensure consistency.

Data Synthesis and Analysis

Given the substantial heterogeneity observed across the included studies, ranging from variations in study designs and biomarker measurement methods to differences in outcome definitions, a meta-analysis was not performed. The primary reasons for this decision were clinical heterogeneity, methodological diversity, and incomplete reporting of effect estimates in some studies. Clinical heterogeneity arose from differing definitions of PE and inconsistent biomarker measurement protocols, making it challenging to pool data meaningfully. Methodological diversity, such as variations in study designs and the extent of adjustment for confounders, further complicated the comparability of results. Additionally, some studies lacked detailed statistical measures necessary for meta-analysis, such as confidence intervals or standard deviations.

A narrative approach was employed to summarize and interpret the findings. This approach allowed for the organization of results by biomarker type, such as cytokines, acute-phase proteins, and oxidative stress markers, and their associations with PE severity or onset. Tables and descriptive summaries were used to compare and contrast findings across studies, highlighting consistencies, discrepancies, and gaps in the evidence. This method provided a comprehensive overview of the current state of knowledge while acknowledging the limitations imposed by the heterogeneity of the included studies.

Ethical Considerations

As this systematic review involved the analysis of previously published data, ethical approval was not required. However, it was ensured that all original studies included in the review had obtained the necessary ethical clearances for their research. This adherence to ethical standards underscores the integrity and reliability of the synthesized evidence.

Results

Search Results

**Figure 1:**
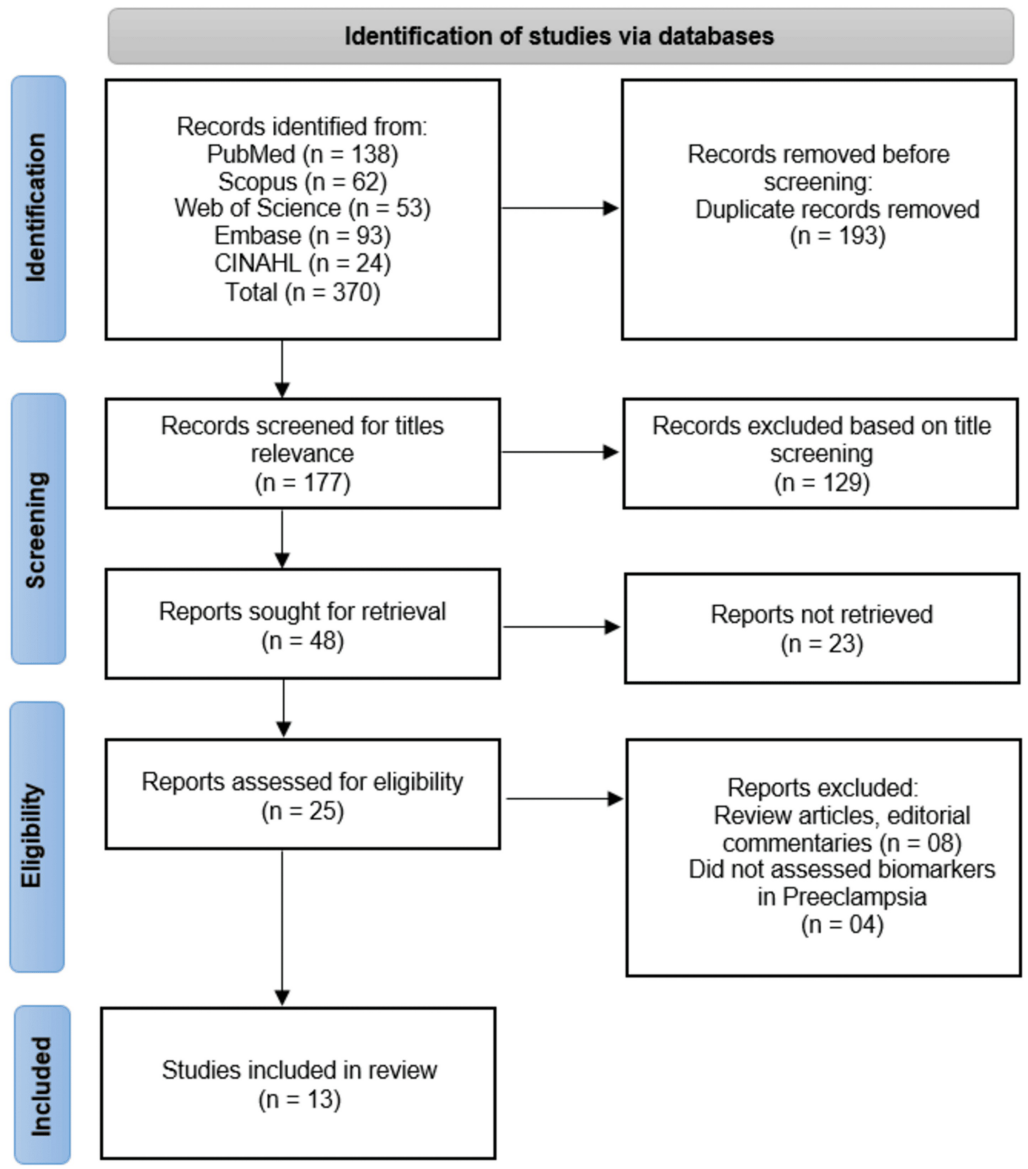
Overview of study screening and inclusion as per the Preferred Reporting Items for Systematic Reviews and Meta-Analyses guidelines.

The systematic search across five databases (PubMed, Scopus, Web of Science, Embase, and CINAHL) initially identified 370 records. After removing 193 duplicate records, 177 studies underwent title screening, of which 129 were excluded for irrelevance. The remaining 48 full-text articles were sought for retrieval, but 23 could not be obtained. Of the 25 reports assessed for eligibility, eight were excluded as review articles or editorial commentaries, and four did not assess biomarkers in PE, leaving 13 studies [14-26] for final inclusion in the systematic review (Figure 1).

Study Characteristics

The systematic review included 13 observational studies [14-26] investigating the association between inflammatory biomarkers and the severity or onset of PE. The studies were conducted across diverse geographical regions, including China [14,21], Turkey [15,18,22,23], Mexico [16], the United States [17], Brazil [19,20], Portugal [24], New Zealand [25], and Hungary [26]. Study designs varied, encompassing case-control, cross-sectional, and prospective cohort studies, with sample sizes ranging from 80 to 466 participants. The study populations predominantly consisted of pregnant women, with cases of PE compared to normotensive controls. Biomarkers assessed included pro-inflammatory cytokines (e.g., IL-6, TNF-α, IL-8), anti-inflammatory cytokines (e.g., IL-10, transforming growth factor-beta (TGF-β)), acute-phase proteins (e.g., CRP), and oxidative stress markers (e.g., malondialdehyde (MDA), thiobarbituric acid reactive substances (TBARS)). Timing of biomarker measurement varied, with some studies collecting samples during pregnancy and others at delivery (Table 1).

**Table 1:** Characteristics of the included studies. BP: blood pressure; BMI: body mass index; CRP: C-reactive protein; D-dimer: fibrin degradation product D-dimer; E-DII: Energy-adjusted Dietary Inflammatory Index; EO: early-onset; FGR: fetal growth restriction; HELLP: hemolysis, elevated liver enzymes, and low platelet count; hs-CRP: high-sensitivity C-reactive protein; ICAM-1: intercellular adhesion molecule 1; IFN-γ: interferon-gamma; IL: interleukin; IL-1RA: interleukin-1 receptor antagonist; IP-10: interferon gamma-induced protein 10; IUGR: intrauterine growth restriction; LO: late-onset; MCP-1: monocyte chemoattractant protein-1; MDA: malondialdehyde; NEO: neopterin; NP: normal pregnancy; PAI-1: plasminogen activator inhibitor-1; PE: preeclampsia; ROC: receiver operating characteristic; SA: sialic acid; sL-selectin: soluble L-selectin; sPECAM: soluble platelet endothelial cell adhesion molecule; suPAR: soluble urokinase plasminogen activator receptor; sVCAM: soluble vascular cell adhesion molecule; TAS: total antioxidant status; TBARS: thiobarbituric acid reactive substances; TGF-β: transforming growth factor-beta; TNF-α: tumor necrosis factor-alpha; VCAM-1: vascular cell adhesion molecule 1

Author (year)	Country	Study design	Sample size (n)	Population characteristics	Biomarker(s) assessed	Timing of biomarker measurement	Outcome assessed	Key findings
Liu et al. [14] (2023)	China	Observational (case-control)	466 PE cases + 466 matched controls	Pregnant women aged >18 years; cases and controls matched by age (±3 years), gestational week (±1 week), and gestational diabetes mellitus	IFN-γ, IL-2, IL-4, TGF-β	During pregnancy	Development of PE	E-DII scores correlated with increased levels of IL-2, IL-4, and TGF-β and higher risk of PE; each unit increase in E-DII score raised PE odds by 30%
Kara et al. [15] (2019)	Turkey	Observational comparative study	80	20 with PE, 24 with IUGR, and 36 healthy pregnant women (controls)	hs-CRP, SA, and IL-6	Fasting blood samples during pregnancy	Presence of PE and IUGR compared to normal pregnancy	No significant elevation of hs-CRP, SA, and IL-6 in PE/IUGR vs. controls; suggested role of local rather than systemic inflammation in pathogenesis
Valencia-Ortega et al. [16] (2019)	Mexico	Cross-sectional	100 (50 PE (30 EO, 20 LO); 50 NP)	Pregnant women: 50 with PE (EO and LO) and 50 with normal pregnancy	TNF-α, IL-6, IL-8, IL-10, IL-1RA, ICAM-1, VCAM-1, pro/anti-inflammatory cytokine ratios	Placental and decidual tissue at delivery; maternal and umbilical serum	Association of biomarkers with PE severity and onset (early vs. late)	PE linked to proinflammatory placental state and elevated maternal VCAM-1; EO PE shows placental proinflammatory markers; LO PE shows systemic maternal inflammation and higher IL-6; no differences in decidual or umbilical concentrations
Ferguson et al. [17] (2017)	USA (from LIFECODES cohort)	Prospective cohort study	441 (50 with PE, 391 normotensive)	Pregnant women enrolled early in gestation; diverse population including higher-risk groups (e.g., African American mothers, higher BMI, preterm birth)	Inflammation markers: C-reactive protein, IL-1β, IL-6, IL-10, TNF-α; oxidative stress markers: 8-isoprostane, 8-hydroxydeoxyguanosine	Four time points during gestation: median 10, 18, 26, and 35 weeks	Onset and severity of PE	Elevated hazard ratios for inflammation biomarkers, especially at 18 weeks; TNF-α consistently elevated across all time points; inflammation and oxidative stress markers varied by gestational age; associations were weaker in higher-risk subgroups; biomarkers alone are not predictive but help characterize the maternal inflammation profile
Cakmak et al. [18] (2016)	Turkey	Cross-sectional	NR	Women with PE and normotensive controls	Endocan, TNF-α	After 20 weeks of gestation	Presence and severity of PE	Serum endocan and TNF-α levels were significantly higher in PE patients than controls; endocan correlated with BP, proteinuria, and TNF-α; higher endocan levels in severe vs. mild PE; ROC analysis supports endocan’s discriminative ability
Pinheiro et al. [19] (2014)	Brazil	Comparative observational study	156 (59 severe PE, 49 normotensive pregnant, and 48 non-pregnant)	Pregnant women with severe PE, normotensive pregnant women, and non-pregnant women	D-dimer, PAI-1, IL-8, IL-6, TNF-α, and IFN-γ	During pregnancy (time not explicitly stated)	Severe PE vs. normotensive pregnancy vs. non-pregnant status	D-dimer and PAI-1 were significantly higher in severe PE; IL-8, IL-6, and IFN-γ were higher in severe PE vs. normotensive pregnancy; D-dimer and PAI-1 showed strong diagnostic performance (AUC); weak correlations were found between some markers
Silva et al. [20] (2013)	Brazil	Case-control	100 (50 PE, 50 healthy pregnant women)	Pregnant women with and without PE	MDA, IL-6, IL-10, TNF-α, and IL-6/IL-10 ratio	During pregnancy	PE status (presence vs. absence)	MDA levels did not differ; IL-6, IL-10, TNF-α, and IL-6/IL-10 ratio were significantly higher in PE women, indicating immune dysfunction
Xiao et al. [21] (2012)	China	Observational comparative study	179 (104 with PE, 75 healthy pregnant women)	Pregnant women with PE (early/late onset, mild/severe, with/without FGR) and healthy gestation-matched controls	IL-6	Maternal circulation during pregnancy	Severity and onset of PE; association with FGR	IL-6 levels were significantly higher in EO and LO PE vs. controls; higher in severe but not mild PE; no correlation with FGR
Ozler et al. [22] (2012)	Turkey	Observational, comparative (cross-sectional)	NR	Pregnant women diagnosed with mild PE, severe PE, HELLP syndrome, and normotensive controls; hospitalized between October 2011 and March 2012	IL-6, TNF-alpha, and NEO	During hospitalization for PE diagnosis	Severity of PE (mild, severe, HELLP)	IL-6 and TNF-alpha levels did not differ significantly among groups (p > 0.05); NEO levels were significantly higher in mild and severe PE and highest in HELLP syndrome; NEO levels correlated with disease severity (r = 0.533, p = 0.000)
Cemgil Arikan et al. [23] (2012)	Turkey	Comparative cross-sectional study	138 (56 normotensive healthy pregnant women, 42 mild PE, 40 severe PE)	Maternal age, gestational age, and BMI matched pregnant women	IL-4, IL-8, IL-12, IFN-γ, and CRP	During pregnancy (exact gestational age not specified)	Severity of PE; correlation with fetal birth weight	IL-8 and CRP were significantly higher in severe PE; IL-12 positively correlated with fetal birth weight in severe PE
Catarino et al. [24] (2012)	Portugal	Comparative observational study	88 (42 healthy pregnant women, 46 PE women, plus their neonates)	Pregnant women with normal pregnancy and PE, and their neonates	Acute-phase proteins (CRP, α1-antitrypsin), proinflammatory cytokines (IL-6, TNF-α), leukocyte activation markers (elastase, lactoferrin, sL-selectin, sVCAM, sPECAM), total antioxidant status (TAS), thiobarbituric acid reactive substances (TBARS), uric acid	Maternal and umbilical cord blood	Association of inflammatory and oxidative stress markers with PE	Higher maternal levels of IL-6, TNF-α, α1-antitrypsin, CRP, sVCAM, uric acid, and TBARS; lower sL-selectin in PE. In newborns of PE mothers: higher uric acid, α1-antitrypsin, CRP; lower leukocyte count, sL-selectin, lactoferrin, elastase/α1-antitrypsin ratio. Suggests an enhanced maternal and fetal inflammatory state linked to endothelial dysfunction and cytokine synthesis
Toldi et al. [25] (2011)	New Zealand	Comparative cross-sectional study	103 (62 healthy pregnant, 41 PE women)	Pregnant women in the third trimester; comparison between healthy and PE groups	suPAR, IL-6, hs-CRP	Third trimester of pregnancy	PE vs. healthy pregnancy	suPAR, IL-6, and hs-CRP levels were significantly higher in PE; suPAR showed a narrower range and was suitable for detecting systemic inflammation in pregnancy
Szarka et al. [26] (2010)	Hungary	Comparative observational study	179 (60 PE patients, 60 healthy pregnant women, 59 healthy non-pregnant women)	Pregnant women with PE, healthy pregnant women, and healthy non-pregnant women	Cytokines (IL-1β, IL-1ra, IL-2, IL-4, IL-6, IL-8, IL-10, IL-12p40, IL-12p70, IL-18, IFN-γ, TNF-α, TGF-β1), chemokines (IP-10, MCP-1), adhesion molecules (ICAM-1, VCAM-1); CRP, von Willebrand factor antigen, fibronectin, malondialdehyde, cell-free fetal DNA	During pregnancy (serum samples)	Severity and systemic inflammatory environment of PE	PE was associated with increased proinflammatory cytokines, chemokines, and adhesion molecules; levels correlated with blood pressure, renal and liver function, and other inflammation markers; findings support a shift to a proinflammatory systemic environment in PE

Inflammatory Biomarkers and Preeclampsia Risk

Several studies reported significant associations between inflammatory biomarkers and the risk of PE. Liu et al. [14] found that higher Dietary Inflammatory Index (E-DII) scores correlated with elevated levels of IL-2, IL-4, and TGF-β, with each unit increase in E-DII raising the odds of PE by 30% (OR = 1.30, 95% CI = 1.18-1.43). Similarly, Ferguson et al. [17] observed elevated hazard ratios for inflammation biomarkers, particularly TNF-α, which was consistently elevated across all gestational time points (HR = 1.49-1.63). Cakmak et al. [18] reported significantly higher serum levels of endocan and TNF-α in PE patients compared to controls (p < 0.001), with endocan levels correlating with disease severity, systolic blood pressure, and proteinuria.

Biomarkers and Disease Severity

The severity of PE was associated with distinct inflammatory profiles. Valencia-Ortega et al. [16] demonstrated that early-onset PE (EO-PE) was linked to a pro-inflammatory placental state, characterized by decreased IL-10 and increased IL-8/interleukin-1 receptor antagonist (IL-1RA) ratios, while late-onset PE (LO-PE) exhibited systemic maternal inflammation with elevated IL-6. Ozler et al. [22] found that neopterin (NEO) levels, measured using enzyme-linked immunosorbent assay, were significantly higher in mild PE (14.1 nmol/L, p = 0.013), severe PE (14.8 nmol/L, p = 0.000), and HELLP syndrome compared to controls, with levels correlating with disease severity (r = 0.533, p = 0.000). Xiao et al. [21] reported that IL-6 levels were significantly higher in EO-PE and LO-PE compared to controls, with further elevations in severe but not mild PE.

Biomarkers and Diagnostic Potential

Some biomarkers demonstrated diagnostic potential for PE. Pinheiro et al. [19] identified D-dimer and plasminogen activator inhibitor-1 (PAI-1) as significantly elevated in severe PE, with strong diagnostic performance (area under the curve (AUC) not specified). Toldi et al. [25] reported higher levels of soluble urokinase plasminogen activator receptor (suPAR), IL-6, and hs-CRP in PE compared to healthy pregnancies (suPAR: 3.18 vs. 2.02 ng/mL, p = 0.0001; IL-6: 5.99 vs. 1.41 pg/mL, p = 0.0001). Szarka et al. [26] highlighted a pro-inflammatory systemic environment in PE, with elevated levels of IL-6, TNF-α, IL-8, and adhesion molecules (intercellular adhesion molecule 1 (ICAM-1), vascular cell adhesion molecule 1 (VCAM-1)), which correlated with clinical markers of disease severity (Table 2).

**Table 2:** Statistical findings from included studies. α1-antitrypsin: alpha-1 antitrypsin; BP: blood pressure; CI: confidence interval; CRP: C-reactive protein; D-dimer: fibrin degradation product D-dimer; E-DII: Energy-adjusted Dietary Inflammatory Index; EO: early-onset; FGR: fetal growth restriction; HELLP: hemolysis, elevated liver enzymes, and low platelet count; hs-CRP: high-sensitivity C-reactive protein; HR: hazard ratio; IQR: interquartile range; ICAM-1: intercellular adhesion molecule 1; IFN-γ: interferon-gamma; IL: interleukin; IL-1RA: interleukin-1 receptor antagonist; IP-10: interferon gamma-induced protein 10; IUGR: intrauterine growth restriction; LO: late-onset; MCP-1: monocyte chemoattractant protein-1; MDA: malondialdehyde; NEO: neopterin; NP: normal pregnancy; OR: odds ratio; PAI-1: plasminogen activator inhibitor-1; PE: preeclampsia; ROC: receiver operating characteristic; SA: sialic acid; sL-selectin: soluble L-selectin; sPECAM: soluble platelet endothelial cell adhesion molecule; suPAR: soluble urokinase plasminogen activator receptor; sVCAM: soluble vascular cell adhesion molecule; TAS: total antioxidant status; TBARS: thiobarbituric acid reactive substances; TGF-β: transforming growth factor-beta; TNF-α: tumor necrosis factor-alpha; VCAM-1: vascular cell adhesion molecule 1

Author (year)	Biomarker(s)	Comparison groups	Effect estimate	95% CI	P-value	Adjusted variables
Liu et al. [14] (2023)	E-DII, IL-2, IL-4, TGF-β	E-DII: highest vs. lowest tertile; per unit increase; IL-2, IL-4, TGF-β: higher vs. lower levels	E-DII tertile OR = 2.18; E-DII per unit OR = 1.30; IL-2 OR = 1.07; IL-4 OR = 1.26; TGF-β OR = 1.17	E-DII tertile 1.52–3.13; E-DII per unit 1.18–1.43; IL-2 1.03–1.11; IL-4 1.03–1.54; TGF-β 1.06–1.29	E-DII P trend <0.001; E-DII per unit <0.001; IL-2, IL-4, TGF-β significant (exact p not specified)	Multivariable adjusted
Kara et al. [15] (2019)	hs-CRP, SA, IL-6	PE and IUGR vs. healthy pregnancies	Higher mean levels in the PE and IUGR groups compared to controls	NR	>0.05 (statistically insignificant)	NR
Valencia-Ortega et al. [16] (2019)	Placental IL-10, IL-1RA (↓); IL-8/IL-1RA ratio (↑); maternal VCAM-1 (↑); EO: IL-10 (↓), IL-8/IL-10 & IL-8/IL-1RA ratios (↑); LO: IL-6 (↑); decidual and umbilical markers (no difference)	PE vs NP; EO vs LO and NP; LO vs EO and NP	PE: proinflammatory placental state; EO: more proinflammatory placenta; LO: higher maternal inflammation; maternal VCAM-1 ↑ in PE; decidual and umbilical levels unchanged	NR	NR	NR
Ferguson et al. [17] (2017)	CRP, IL-1β, IL-6, IL-10, TNF-α, 8-isoprostane	Preeclamptic vs. normotensive pregnancies (LIFECODES cohort; 50 vs. 391 mothers)	HR 1.31–1.83 for inflammation biomarkers at 18 weeks; HR 1.49–1.63 for TNF-α at all visits; HR 1.68 for 8-isoprostane at 18 weeks	Example: 8-isoprostane 1.14–2.48; others not specified individually	<0.01	Models adjusted; sensitivity analyses for race, BMI, and preterm birth
Cakmak et al. [18] (2016)	Endocan, TNF-α	PE patients vs. normotensive controls; subgroup: severe vs. mild PE; correlation with BP, proteinuria, TNF-α	Endocan: 20.04 (12.26) vs. 15.55 (6.19) ng/mL; TNF-α: 26.49 ± 12.14 vs. 14.62 ± 5.61 pg/mL; Endocan positively correlated with systolic BP (r = 0.618), diastolic BP (r = 0.608), 24-h proteinuria (r = 0.786), TNF-α (r = 0.474); higher in severe PE than mild PE	NR	All p < 0.001	NR
Pinheiro et al. [19] (2014)	D-dimer, PAI-1, IL-8, IL-6, TNF-α, IFN-γ	sPE vs. normotensive pregnant women; sPE vs. non-pregnant women	D-dimer and PAI-1 higher in sPE with good ROC discrimination; IL-8, IL-6, IFN-γ higher in sPE; weak correlations: D-Dimer–IL-8, PAI-1–IFN-γ	NR	NR	NR
Silva et al. [20] (2013)	MDA, IL-6, IL-10, TNF-α, IL-6/IL-10 ratio	PE women (n = 50) vs. healthy pregnant women (n = 50)	MDA: no difference; IL-6, IL-10, TNF-α, IL-6/IL-10 ratio: higher in preeclamptic group	NR	MDA: p > 0.05; others: significant (exact p not given)	NR
Xiao et al. [21] (2012)	IL-6	EO PE vs. healthy; LO PE vs. healthy; Severe PE vs. healthy; mild PE vs. healthy; PE with FGR vs. PE without FGR	IL-6 significantly increased in early onset, late onset, and severe PE; not increased in mild PE; no difference between PE with and without FGR	NR	NR	NR
Ozler et al. [22] (2012)	TNF-alpha, IL-6, NEO	Normotensive controls vs. Mild PE vs. severe PE vs. HELLP syndrome	TNF-alpha and IL-6: no significant difference (p > 0.05); NEO higher in mild PE (14.1 nmol/L, p = 0.013) and severe PE (14.8 nmol/L, p = 0.000) vs. control (10.3 nmol/L); NEO correlated with severity (r = 0.533, p = 0.000); highest in HELLP	NR	TNF-alpha and IL-6: >0.05; NEO: 0.013 (mild PE), 0.000 (severe PE), correlation p = 0.000	NR
Cemgil Arikan et al. [23] (2012)	IL-8, CRP, IL-4, IL-12, IFN-γ	Group 1 (normotensive) vs. Group 2 (mild PE) vs. Group 3 (severe PE)	IL-8 and CRP higher in severe PE vs. controls (p < 0.05); mild PE higher than controls but NS; IL-4, IL-12, IFN-γ no significant difference; IL-12 positively correlated with fetal birth weight in severe PE (p < 0.05)	NR	See the effect estimate	Maternal age, gestational age, and BMI
Catarino et al. [24] (2012)	IL‐6, TNF‐α, α1‐antitrypsin, CRP, sVCAM, sPECAM, sL‐selectin, lactoferrin, elastase/α1‐antitrypsin ratio, uric acid, TBARS, TAS	PE pregnant women vs. normotensive pregnant women; PE newborns vs. normotensive newborns	IL‐6, TNF‐α, α1‐antitrypsin, CRP, sVCAM, uric acid, TBARS: higher in PE; sL‐selectin, lactoferrin, elastase/α1‐antitrypsin ratio: lower in PE	NR	Significant	NR
Toldi et al. [25] (2011)	suPAR, IL-6, hs-CRP	PE vs. healthy pregnancy	suPAR: 3.18 vs. 2.02 ng/mL (IQR 2.30–4.71 vs. 1.81–2.40); IL-6: 5.99 vs. 1.41 pg/mL (IQR 2.97–18.12 vs. 1.00–2.70); hs-CRP: 6.60 vs. 3.90 mg/L (IQR 3.55–15.40 vs. 2.10–7.25)	Not reported as 95% CI, IQR reported instead	suPAR: 0.0001; IL-6: 0.0001; hs-CRP: 0.006	NR
Szarka et al. [26] (2010)	IL-1β, IL-1ra, IL-2, IL-4, IL-6, IL-8, IL-10, IL-12p40, IL-12p70, IL-18, IFN-γ, TNF-α, TGF-β1, IP-10, MCP-1, ICAM-1, VCAM-1	Preeclamptic vs. Healthy Pregnant vs. Healthy Non-pregnant	Pro-inflammatory cytokines (IL-6, TNF-α, IL-8, IP-10, MCP-1, ICAM-1, VCAM-1) increased in preeclampsia; IL-1ra, TNF-α, and MCP-1 decreased in healthy pregnancy vs. non-pregnant; IL-10 decreased, and IP-10 increased in healthy pregnancy	NR	Significant	Correlated with BP, renal/liver function, CRP, malondialdehyde, von Willebrand factor antigen, fibronectin

Maternal and Fetal Inflammatory Responses

Studies also explored maternal-fetal inflammatory dynamics. Catarino et al. [24] found higher maternal levels of IL-6, TNF-α, and CRP in PE, alongside elevated oxidative stress markers (e.g., TBARS). These disturbances were mirrored in newborns of PE mothers, who exhibited higher uric acid and CRP levels, suggesting a shared inflammatory state. Similarly, Cemgil Arikan et al. [23] reported that IL-12 levels in severe PE positively correlated with fetal birth weight (p < 0.05), indicating a potential link between maternal inflammation and fetal outcomes.

Inconsistent Findings

Not all studies reported consistent associations. Kara et al. [15] found no significant elevation in hs-CRP, sialic acid, or IL-6 in PE or intrauterine growth restriction (IUGR) compared to controls (p > 0.05), suggesting a possible role for local rather than systemic inflammation. Silva et al. [20] observed no difference in MDA levels between PE and controls but reported higher IL-6, IL-10, and TNF-α in PE, indicating immune dysfunction without oxidative stress involvement.

Quality Assessment Results

Seven studies demonstrated low risk of bias [14,16,17,21,24-26], scoring 7-9 stars due to robust study designs, representative samples, and adequate adjustment for confounders. The remaining six studies [15,18-20,22,23] were rated as moderate risk (5-6 stars), primarily due to limitations such as insufficient control for confounding variables, small sample sizes, or unclear exposure assessment timing. No studies were classified as high risk. While the majority of findings were supported by methodologically sound research, the moderate-risk studies warrant cautious interpretation, particularly where key covariates such as BMI or gestational age were unaccounted for [15,22] or where biomarker measurement timing was inconsistent [19,20]. Overall, the evidence base remains largely reliable, though heterogeneity in study quality underscores the need for standardized methodologies in future research (Table 3).

**Table 3:** Quality assessment of included studies using Newcastle-Ottawa Scale risk of bias tool.

Author (year)	Selection (Max 4★)	Comparability (Max 2★)	Outcome (Max 3★)	Total score (Max 9★)	Risk of bias
Liu et al. [14] (2023)	★★★★	★★	★★★	9	Low
Kara et al. [15] (2019)	★★★	★	★★	6	Moderate
Valencia-Ortega et al. [16] (2019)	★★★★	★★	★★	8	Low
Ferguson et al. [17] (2017)	★★★★	★★	★★★	9	Low
Cakmak et al. [18] (2016)	★★★	★	★★	6	Moderate
Pinheiro et al. [19] (2014)	★★★	★	★★	6	Moderate
Silva et al. [20] (2013)	★★★	★	★★	6	Moderate
Xiao et al. [21] (2012)	★★★★	★★	★★	8	Low
Ozler et al. [22] (2012)	★★★	★	★★	6	Moderate
Cemgil Arikan et al. [23] (2012)	★★★	★	★★	6	Moderate
Catarino et al. [24] (2012)	★★★★	★★	★★	8	Low
Toldi et al. [25] (2011)	★★★★	★★	★★	8	Low
Szarka et al. [26] (2010)	★★★★	★★	★★	8	Low

Discussion

The findings of this systematic review underscore the significant role of inflammatory biomarkers in the pathogenesis, severity, and onset of PE, while also highlighting inconsistencies and methodological variations across studies. The synthesis of 13 observational studies reveals a complex interplay between systemic and localized inflammatory responses, endothelial dysfunction, and oxidative stress, which collectively contribute to the clinical manifestations of PE. The majority of included studies [14,17,21,26] consistently reported elevated levels of pro-inflammatory cytokines, such as IL-6, TNF-α, and IL-8, in women with PE compared to normotensive controls. These findings align with existing literature that characterizes PE as a pro-inflammatory state, where aberrant placental development and immune dysregulation trigger systemic endothelial damage [27,28]. For instance, the repeated measures design by Ferguson et al. [17] demonstrated that TNF-α remained elevated throughout gestation in PE women, suggesting sustained inflammation as a hallmark of the disease. This is further supported by Szarka et al. [26], who identified a broader cytokine and chemokine dysregulation, including elevated IP-10 and MCP-1, which are known to exacerbate endothelial dysfunction [29].

The association between inflammatory biomarkers and PE severity was particularly evident in studies stratifying cases by onset (early vs. late) or clinical severity (mild vs. severe). Valencia-Ortega et al. [16] reported distinct inflammatory profiles for EO-PE and LO-PE, with EO-PE characterized by placental pro-inflammatory markers (e.g., decreased IL-10, elevated IL-8/IL-1RA ratios) and LO-PE associated with systemic maternal inflammation (e.g., higher IL-6). These findings corroborate the "two-stage model" of PE, where EO-PE arises from placental malperfusion and LO-PE reflects maternal systemic dysfunction [30]. Similarly, Ozler et al. [22] identified NEO as a biomarker correlating with disease severity, with levels highest in HELLP syndrome, suggesting its potential utility in risk stratification. This aligns with prior work by Verlohren et al. [31], who proposed that biomarkers such as soluble fms-like tyrosine kinase-1 (sFlt-1) and placental growth factor (PlGF) could differentiate PE subtypes. However, the inconsistent performance of some biomarkers, such as hs-CRP and IL-6 in Kara et al. [15], challenges the universality of systemic inflammation in PE and points to the role of localized placental inflammation, as hypothesized by Harmon et al. [32].

The diagnostic potential of inflammatory biomarkers was explored in several studies, with mixed results. Pinheiro et al. [19] and Toldi et al. [25] reported strong discriminative performance for D-dimer, PAI-1, and suPAR, respectively, in identifying severe PE. These findings are promising but must be contextualized within the limitations of single-center studies and variability in assay methods. For example, while suPAR demonstrated a narrow range in healthy pregnancies [25], its clinical utility as a standalone marker remains uncertain without validation in larger, diverse cohorts. Similarly, the correlation between endocan and disease severity [18] introduces a novel biomarker linked to endothelial dysfunction, yet its pathophysiological role warrants further investigation. Comparatively, existing literature emphasizes multi-marker panels (e.g., combining sFlt-1, PlGF, and soluble endoglin) for improved PE prediction [33], suggesting that future research should explore integrative models incorporating inflammatory and angiogenic markers.

Maternal-fetal inflammatory responses were another critical theme, with Catarino et al. [24] and Cemgil Arikan et al. [23] demonstrating that maternal inflammation extends to the fetal compartment, as evidenced by elevated uric acid and CRP in neonates of PE mothers. This mirrors earlier findings by Mulla et al. [34], who reported fetal immune activation in PE, potentially predisposing offspring to long-term metabolic and cardiovascular risks [35]. The positive correlation between IL-12 and fetal birth weight in severe PE [23] further suggests that cytokine profiles may modulate fetal growth, though the exact mechanisms remain unclear. These observations reinforce the concept of PE as a multisystem disorder with intergenerational implications, as highlighted by Roberts and Hubel [36].

Despite these advances, inconsistencies in biomarker associations warrant caution. For instance, Kara et al. [15] found no significant differences in hs-CRP or IL-6 between PE and controls, contradicting the meta-analysis by Lau et al. [37], which reported CRP as a reliable inflammatory marker in PE. Similarly, Silva et al. [20] observed no oxidative stress (MDA) differences but noted immune dysregulation (elevated IL-6/IL-10 ratio), underscoring the heterogeneity of PE phenotypes. Such discrepancies may arise from variations in study design, sample timing, or population characteristics (e.g., BMI, ethnicity), as noted in the LIFECODES cohort [17], where biomarker associations were attenuated in high-risk subgroups. This heterogeneity echoes the call by Stepan et al. [38] for standardized protocols in PE biomarker research to enhance comparability.

Limitations

This review has several limitations. First, the predominance of observational designs precludes causal inferences, and residual confounding (e.g., unmeasured sociodemographic or genetic factors) may bias associations. Second, heterogeneity in biomarker measurement timing (e.g., trimester-specific vs. delivery samples) complicates cross-study comparisons, as highlighted by the variability in IL-6 findings [21,22]. Third, the exclusion of non-English studies and gray literature may introduce selection bias. Finally, the moderate risk of bias in six studies [15,19] due to inadequate confounder adjustment or small samples necessitates cautious interpretation.

## Conclusions

This review consolidates robust evidence linking inflammatory biomarkers to PE pathogenesis, severity, and onset, while also revealing critical knowledge gaps. Pro-inflammatory cytokines (e.g., IL-6, TNF-α), endothelial markers (e.g., endocan, suPAR), and oxidative stress mediators collectively underscore PE’s systemic inflammatory nature. However, inconsistent findings and methodological disparities highlight the need for larger, standardized studies integrating multi-omics approaches to refine biomarker panels for early prediction and personalized management. Future research should prioritize longitudinal designs, diverse populations, and mechanistic studies to unravel the causal pathways underlying inflammation in PE, ultimately informing targeted therapeutic strategies.

## Appendices

**Table 4:** Search strategy.

Database	Search strategy
PubMed	(“preeclampsia”[MeSH Terms] OR “preeclampsia”[Title/Abstract] OR “pre-eclampsia”[Title/Abstract]) AND (“biomarkers”[MeSH Terms] OR “biomarker*”[Title/Abstract]) AND (“inflammation”[MeSH Terms] OR “inflammation”[Title/Abstract] OR “inflammatory”[Title/Abstract]) AND (“severity”[Title/Abstract] OR “onset”[Title/Abstract] OR “early-onset”[Title/Abstract] OR “late-onset”[Title/Abstract])
Scopus	(TITLE-ABS-KEY (preeclampsia OR “pre-eclampsia”) AND TITLE-ABS-KEY (biomarker*) AND TITLE-ABS-KEY (inflammation OR inflammatory) AND TITLE-ABS-KEY (severity OR onset OR “early-onset” OR “late-onset”))
Web of Science	TS=(preeclampsia OR “pre-eclampsia”) AND TS=(biomarker*) AND TS=(inflammation OR inflammatory) AND TS=(severity OR onset OR “early-onset” OR “late-onset”)
Embase	(‘preeclampsia’/exp OR preeclampsia:ti,ab OR ‘pre-eclampsia’:ti,ab) AND (‘biomarker’/exp OR biomarker*:ti,ab) AND (‘inflammation’/exp OR inflammation:ti,ab OR inflammatory:ti,ab) AND (severity:ti,ab OR onset:ti,ab OR ‘early-onset’:ti,ab OR ‘late-onset’:ti,ab)
CINAHL	(MH “Preeclampsia” OR preeclampsia OR “pre-eclampsia”) AND (MH “Biomarkers” OR biomarker*) AND (MH “Inflammation” OR inflammation OR inflammatory) AND (severity OR onset OR “early-onset” OR “late-onset”)
